# Slug Is a Predictor of Poor Prognosis in Esophageal Squamous Cell Carcinoma Patients

**DOI:** 10.1371/journal.pone.0082846

**Published:** 2013-12-18

**Authors:** Md. Raghibul Hasan, Rinu Sharma, Anoop Saraya, Tushar K. Chattopadhyay, Siddartha DattaGupta, Paul G. Walfish, Shyam S. Chauhan, Ranju Ralhan

**Affiliations:** 1 Department of Biochemistry, All India Institute of Medical Sciences, New Delhi, India; 2 School of Biotechnology, Guru Gobind Singh Indraprastha University, Delhi, India; 3 Department of Gastroenterology, All India Institute of Medical Sciences, New Delhi, India; 4 Department of Gastrointestinal Surgery, All India Institute of Medical Sciences, Ansari Nagar, New Delhi, India; 5 Department of Pathology, All India Institute of Medical Sciences, New Delhi, India; 6 Department of Medicine, Endocrine Division, Mount Sinai Hospital and University of Toronto, Toronto, Ontario, Canada; 7 Alex and Simona Shnaider Research Laboratory in Molecular Oncology, Department of Pathology & Laboratory Medicine, Mount Sinai Hospital, Toronto, Ontario, Canada; 8 Department of Pathology and Laboratory Medicine, Mount Sinai Hospital, Toronto, Ontario, Canada; 9 Joseph and Mildred Sonshine Family Centre for Head and Neck Diseases, Department of Otolaryngology, Head and Neck Surgery, Mount Sinai Hospital, Toronto, Ontario, Canada; 10 Department of Otolaryngology, Head and Neck Surgery, University of Toronto, Toronto, Ontario, Canada; Vanderbilt University Medical Center, United States of America

## Abstract

**Background:**

Slug, a regulator of epithelial mesenchymal transition, was identified to be differentially expressed in esophageal squamous cell carcinoma (ESCC) using cDNA microarrays by our laboratory. This study aimed to determine the clinical significance of Slug overexpression in ESCC and determine its correlation with clinicopathological parameters and disease prognosis for ESCC patients.

**Methods:**

Immunohistochemical analysis of Slug expression was carried out in archived tissue sections from 91 ESCCs, 61 dysplastic and 47 histologically normal esophageal tissues. Slug immunopositivity in epithelial cells was correlated with clinicopathological parameters and disease prognosis over up to 7.5 years for ESCC patients.

**Results:**

Increased expression of Slug was observed in esophageal dysplasia [cytoplasmic, 24/61 (39.3%) cases, p = 0.001, odd’s ratio (OR) = 4.7; nuclear, 11/61 (18%) cases, p < 0.001, OR = 1.36] in comparison with normal esophageal tissues. The Slug expression was further increased in ESCCs [cytoplasmic, 64/91 (70.3%) p < 0.001, OR = 10.0; nuclear, 27/91 (29.7%) p < 0.001, OR = 1.42]. Kaplan Meier survival analysis showed significant association of nuclear Slug accumulation with reduced disease free survival of ESCC patients (median disease free survival (DFS) = 6 months, as compared to those that did not show overexpression, DFS = 18 months; p = 0.006). In multivariate Cox regression analysis nuclear Slug expression [p= 0.005, Hazard’s ratio (HR) = 2.269, 95% CI = 1.289 - 3.996] emerged as the most significant independent predictor of poor prognosis for ESCC patients.

**Conclusions:**

Alterations in Slug expression occur in early stages of development of ESCC and are sustained during disease progression. Slug may serve as a diagnostic biomarker and as a predictor of poor disease prognosis to identify ESCC patients that are likely to show recurrence of the disease.

## Introduction

Epithelial to mesenchymal transition (EMT) is a dynamic cellular process by which cells lose cell-cell junctions and baso-apical polarity acquiring mesenchymal characteristics with increased motility and invasive potential, stemlike characteristics and resistance to apoptosis that are essential for the development of metastatic disease. Major signaling pathways involved in EMT include Ras, transforming growth factor-β (TGF-β), Wnt, epidermal growth factor (EGF), Notch and Hedgehog. These signalling pathways activated by extrinsic or intrinsic stimuli will ultimately converge on any of the transcription factors, including nuclear factor kappa B (NFκB) and zinc finger proteins Snail and Slug, Twist, ZEB 1/2, and Smads that will likely culminate in transcriptional repression of *E-cadherin*. These transcription factors interact with one another and other proteins to provide crosstalk between the relevant signalling pathways and regulate the phenotypic changes of cancer cells. EGF and TGF-β promote EMT by regulating Snail, Twist, and Slug through direct regulation of genes involved in cellular adhesion, migration, and invasion [1-3]. Thus these EMT regulators may play an important role in cancer progression. Snail and Slug have been described as direct repressors of *E-cadherin* and inducers of EMT and invasion when overexpressed in epithelial cells [1-3]. However, the role of these transcriptional regulators in early premalignant stages such as dysplasia prior to development of frank malignancy remains yet unknown.

The clinical relevance of Slug expression has been demonstrated in several human cancers including breast, prostate, head and neck, pancreas and endometrial carcinomas [4-8]. Slug expression showed stronger correlation with loss of E-cadherin in breast cancer cell lines than did SNAIL expression suggesting Slug is a likely in vivo repressor of E-cadherin expression in breast cancer [9,10]. Expression of Slug especially in the E-cadherin preserved tumors has been shown to be related to prognosis in esophageal cancer [11-13]. 

Esophageal cancer is the eighth most common cancer worldwide, with 4,82,000 new cases (3.8% of the total) estimated in 2008, and the sixth most common cause of death from cancer with 4,07,000 deaths (5.4% of the total)[14,15]. It has extremely poor prognosis owing to insidious symptomatology, late clinical presentation and rapid progression [16,17]. Despite advances in multimodality therapy, due to late stage of diagnosis and poor efficacy of treatment, the prognosis for patients with esophageal squamous cell carcinoma (ESCC) still remains poor with an average 5-year survival of less than 30% globally [18,19]. Development of better preventive approaches and more effective treatment modalities requires in-depth understanding of molecular mechanisms involved in the complex process of esophageal carcinogenesis. There is an urgent need for identification of novel molecular markers to provide the clinician with useful information concerning patient prognosis and possible therapeutic options [20-22]. In search of molecular markers our laboratory analyzed global gene expression profiles of ESCCs using commercially available 19.1k cDNA microarrays [23]. One of the salient findings was the identification of 19 differentially expressed genes encoding zinc binding or modulating proteins; Slug, a key transcription factor regulating EMT was one of these proteins found to be overexpressed in ESCCs [23]. Slug expression has been shown to be related to prognosis in esophageal cancer [11-13]. However, the clinical impact of Slug expression in early stages of esophageal cancer development remains yet unknown.

Here in we focussed on understanding the clinical relevance of Slug in esophageal carcinogenesis and progression of esophageal cancer. The aim of the present study was to examine the clinical significance of Slug expression in early stages of esophageal cancer development namely esophageal dysplasia and in frank malignancy (ESCC). Slug expression was also correlated with clinicopathological parameters of ESCC patients and with disease prognosis. 

## Materials and Methods

### Patients and clinicopathological data collection, tissue specimens

This study was approved by the All India Institute of Medical Sciences (AIIMS) Research Ethics Board, New Delhi, India prior to its commencement. Written informed consent was obtained for the acquisition and use of patient tissue samples and anonymized clinical data. Tissue specimens were obtained by diagnostic or therapeutic procedures from 61 patients with clinically defined esophageal dysplasia attending the Outpatient Clinic of the Departments of Surgical Disciplines and Gastrointestinal Surgery, AIIMS, and from 91 ESCC patients undergoing curative cancer surgery during the period 2005 - 2010, after obtaining the patients’ written consent. Wherever possible, non-malignant tissues (n = 47) were taken each from a site distant from the surgically resected ESCC, or collected from the patients attending the Endoscopy clinic in the Outpatient Department of Gastroenterology. Taken together, these 47 non-malignant esophageal tissues with histological evidence of normal epithelia constituted the normal group. After excision, tissues were immediately snap-frozen in liquid nitrogen and stored at -80°C in the Research Tissue Bank till further use; one part of the tissue was collected in 10% formalin and embedded in paraffin for histopathological and immunohistochemical analyses. Histologically confirmed esophageal normal epithelia, dysplasia, and ESCC as revealed by H & E staining were used for immunohistochemistry [24]. Patient demographic, clinical, and pathological data were recorded in a pre-designed Performa as described previously [24]. The information documented included clinical TNM staging (tumor, node, and metastasis based on the Union International Center le Cancer TNM classification of malignant tumors 2002), site of the lesion, histopathological differentiation, age, gender, and tobacco consumption habits.

### Follow-up Study

Eighty two of 91 ESCC patients who underwent treatment from 2005–2010 could be investigated and evaluated in the esophageal cancer follow-up clinic at regular time intervals, while 9 patients did not report in the follow up clinic. Survival status of the ESCC patients was verified and updated from the records of the Tumor Registry, Department of Gastrointestinal Surgery, AIIMS, as of June 2013. ESCC patients were monitored for a maximum period of 7.5 years. Disease-free survival time is defined as the time from completion of primary treatment till the patient showed any clinical and radiological evidence of local or regional disease, or distant metastasis at the time of the last follow-up of patients monitored in this study. Twenty eight patients who did not show recurrence were alive until the end of the follow-up period. Only disease-free survival was evaluated in the present study, as the number of deaths due to disease progression did not allow a reliable statistical analysis. Disease-free survival was expressed as the number of months from the date of surgery to loco-regional relapse/death.

### Immunohistochemistry

Paraffin-embedded sections (5 µm) of human esophageal histological normal (n = 47), dysplasia (n = 61) and ESCC (n = 91) were collected on gelatin-coated slides. The ESCC tissues analysed in this study had more than 80% tumor cells in H&E sections. In brief, the sections were deparaffinized in xylene, hydrated in gradient alcohol, and pre-treated in a microwave oven for 10 min at 800 W and 5 min at 480 W in Tris-EDTA (10mM Tris, 1mM EDTA, pH = 9.0) for antigen retrieval. The sections were incubated with hydrogen peroxide (3% v/v) in methanol for 30 min to quench the endogenous peroxidise activity, followed by blocking with 1% bovine serum albumin (BSA) to preclude nonspecific binding. Thereafter, the slides were incubated with rabbit polyclonal anti-Slug antibody (0.5 mg/ml, sc-15391, Santa Cruz Biotechnology, CA) for 16 h at 4°C. The primary antibody was detected using the streptavidin-biotin complex with the Dako LSAB plus kit (Dako Cytomation, Glostrup, Denmark) and diaminobenzidine as the chromogen as described before [24]. In the negative control tissue sections, the primary antibody was replaced by isotype specific non-immune mouse IgG. A section from breast cancer tissue was used as a positive control in each batch of immunohistochemistry.

### Evaluation of immunohistochemical staining

 Each tissue section was evaluated for Slug immunostaining using a semi-quantitative scoring system for both staining intensity and the percentage of positive epithelial cells [24]. For Slug protein expression, sections were scored as positive if epithelial cells showed immunopositivity in the nucleus/cytoplasm when observed independently by three of us (MRH, RS, SDG), who were blinded to the clinical outcome (the slides were coded and the scorers did not have prior knowledge of the local tumor burden, lymphonodular spread, and grading of the tissue samples). The tissue sections were scored based on the % of immunostained cells as: 0–10%= 0; 10–30% = 1; 31–50% = 2; 51–70% = 3 and >70% = 4. Sections were also scored semi-quantitatively on the basis of staining intensity as negative = 0; mild = 1; moderate = 2; intense =3 [25]. Finally, a total score was obtained by adding the score of percentage positivity and intensity. In cases where both nuclear and cytoplasmic immunoreactivity was observed, the nuclear and cytoplasmic staining was scored independently. The scoring by the three observers was discrepant in about 5% cases and a consensus on the final result was reached by re-evaluation of these slides and discussion. 

### Statistical Analyses

The immunohistochemical data were subjected to statistical analyses using the SPSS 10.0 software (Chicago, Il). Sensitivity and specificity were calculated and quantified using receiver operating characteristic (ROC) analyses. The positive predictive value (PPV) describes the proportion of the correctly classified cases. Based on sensitivity and specificity values for Slug, a cut-off, 2 was defined as positive criterion for cytoplasmic staining and for nuclear staining a score of 4 was defined as the cut-off for Slug immunopositivity for statistical analyses. The relationships between Slug protein expression and clinicopathological parameters were tested using Chi-Square and Fischer’s exact test. Two-sided p values were calculated and p value < 0.05 was considered to be significant. The correlation of Slug staining with patient survival was evaluated using life tables constructed from survival data with Kaplan-Meier plots. Multivariate analysis was carried out using Cox regression model. 

## Results

### Immunohistochemical analysis of Slug expression in esophageal normal tissues, dysplasia and cancer

**Figure 1:**
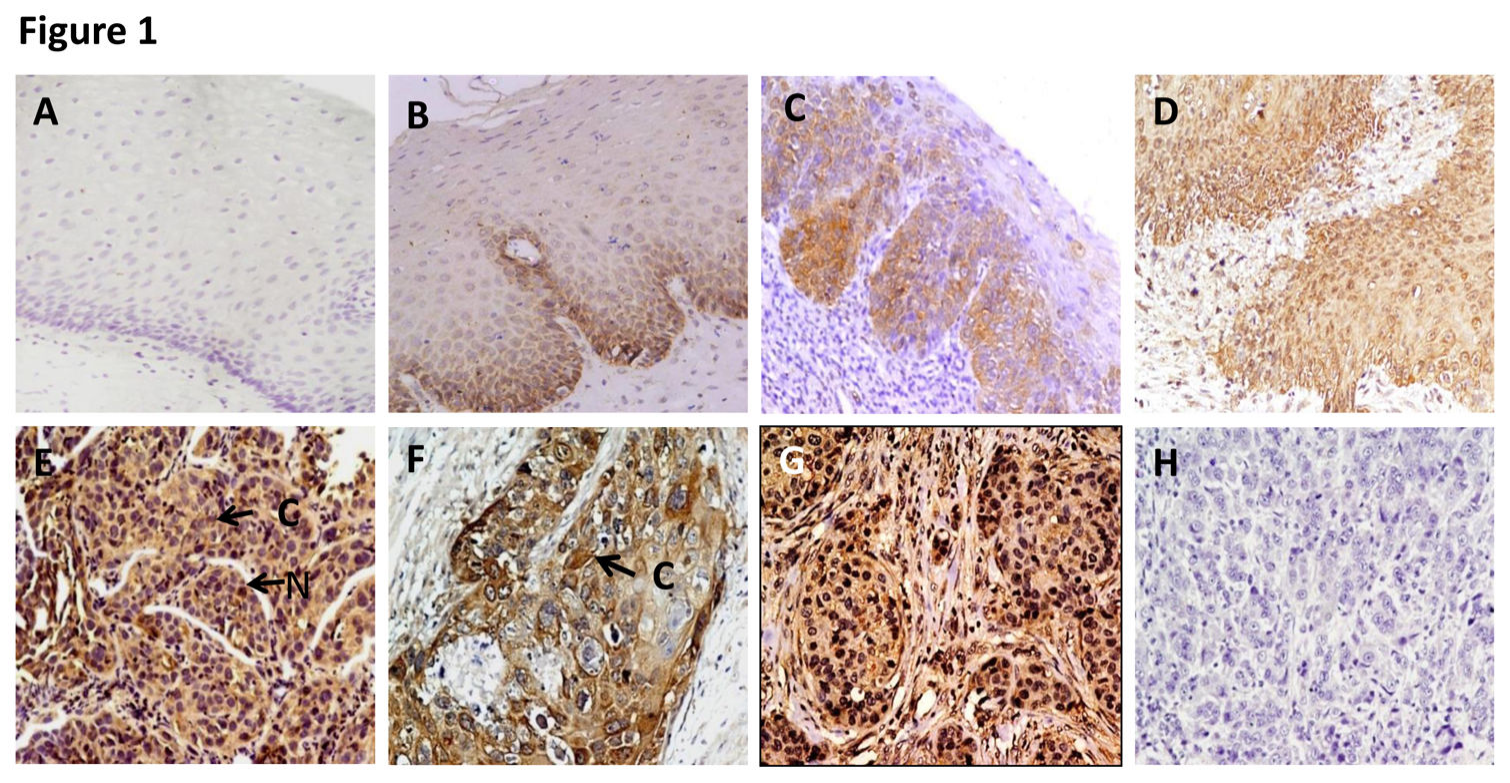
Immunohistochemical analysis of Slug in esophageal tissues. Paraffin-embedded sections of histological normal mucosa, dysplasia, and ESCC were stained using anti-Slug polyclonal antibody as described in the Methods section. (A) Normal esophageal mucosa showing no Slug immunostaining; (B) and (C) Mild and moderate dysplasia depicting low level of nuclear and cytoplasmic Slug immunostaining in epithelial cells respectively; (D) Severe dysplasia shows high expression of nuclear and cytoplasmic Slug; (E) and (F) ESCC illustrating both intense cytoplasmic and nuclear staining in tumor cells; (G) breast cancer tissue used as a positive control showing Slug immunostaining; and (H) ESCC used as a negative control, showing no Slug immunostaining in tumor cells; Arrows show nuclear and cytoplasmic localization (A-E,G,H original magnification x 200; F original magnification x 400).

To determine the clinical significance of Slug protein in esophageal cancer, its expression was analysed in clinical specimens from histologically normal esophageal tissues, dysplasia, and ESCC using a specific anti-Slug antibody by immunohistochemistry. Of the 47 normal tissues analysed, 81% (38/47) did not show detectable Slug immunostaining in nucleus/cytoplasm of the epithelial cells (Figure 1A). In the remaining 9 of 47 (19%) normal tissues, moderate cytoplasmic staining was observed in differentiated epithelial cells in the basal layer only. Chi square trend analysis showed significant increase in Slug expression (nuclear/cytoplasmic) in tissues obtained from different stages of esophageal tumorigenesis (normal, dysplasia and ESCC; Table 1 p_trend_ < 0.001). 

**Table 1:** Immunohistochemical analysis of Slug expression in esophageal tissues and their correlation with clinicopathological parameters.

**Tissue type**	**Total cases (N)**	**Cytoplasmic Positivity**		p-value*	**O.R.**	**(95% CI)**	**Nuclear Positivity**		p-value**	**O.R.**	**(95% CI**)
		**n**	**(%)**				**n**	**(%)**			
**Normal**	47	9	(19)	--	--	--	--	--	--	--	--
**Dysplasia**	61	24	(39.3)	**0.001^*a*^**	**4.7**	**(1.8-10.9)**	11	(18)	**<0.001^*b*^**	**1.36**	**(1.16-1.58)**
Mild	47	12	(25.5)	**<0.001^*c*^**			4	(8.5)	**0.001^*d*^**		
Moderate	8	6	(75)				3	(37.5)			
Severe	6	6	(100)	**<0.001^*e*^**	**4.20**	**(2.10-8.37)**	4	(66.7)	**0.008^*f*^**	**2.90**	**(1.30-6.46)**
**ESCC**	91	64	(70.3)	**<0.001^*g*^**	**10**	**(4.5-23.5)**	27	(29.7)	**<0.001^*h*^**	**1.42**	**(1.24-1.62)**
**Gender**											
Male	60	40	(66.7)				15	(25)			
Female	31	24	(77.4)	0.2	1.7	(0.63-4.6)	12	(38.7)	0.133	1.9	(0.75-4.8)
**Age (years**)											
< 54	42	32	(76.2)				13	(31)			
> 54	49	32	(65.3)	0.18	0.59	(0.23-1.5)	14	(28.6)	0.49	0.9	(0.36-2.2)
**Tumor Stage**											
(T_1_+T_2_)	10	7	(70)				1	(10)			
(T_3_+T_4_)	81	57	(70.3)	0.61	1.0	(0.24-4.2)	26	(32.1)	0.13	4.2	(0.5-35.3)
**Nodal status**											
N_0_	27	19	(70.3)				6	(22.2)			
N_1-4_	64	45	(70.3)	0.60	1.0	(0.37-2.7)	21	(30.3)	0.226	1.7	(0.6-4.8)
**Histological differentiation**											
WDSCC	28	22	(78.6)				9	(32.1)			
MDSCC+PDSCC	63	48	(66.7)	0.18	0.5	(0.2-1.5)	18	(28.6)	0.45	0.86	(0.3-2.2)
**Tobacco habits**											
Non-smoker	38	27	(71)	0.54	--	--	14	(36.8)			
Smoker	53	37	(69.8)				13	(24.5)	0.1	--	--

Parameters: **Normal vs dysplasia** : ^a^cytoplasmic slug , ^b^Nuclear slug ,Chi Square analysis **Grade of dysplasia**: **^*c*^**cytoplasmic slug , ^d^Nuclear slug, Chi Square trend analysis **Dysplasia vs. ESCC**: : ^e^cytoplasmic slug , ^f^Nuclear slug ,Chi Square analysis **Normal vs. ESCC**: : **^*g*^**cytoplasmic slug , ^h^Nuclear slug ,Chi Square analysis

Notably, increased cytoplasmic localization of Slug was observed in 39.3% dysplasia (24 of 61 cases) (p = 0.001, OR = 4.7, 95% CI = 1.8-10.9) in comparison with normal tissues (Table 1and Figure 1B). Similarly, progressive increase in nuclear expression of Slug was also observed in 11/61 (18%) dysplasia (p < 0.001, OR = 1.36, 95%, CI =1.16 - 1.58). There was a significant increase in Slug expression with grade of dysplasia. Increase in cytoplasmic Slug expression was observed in 12/47 (25.5%) mild dysplasia (Figure 1B), 6/8 (75%) moderate dysplasia (Figure 1C), and 6/6 severe dysplasia (Figure 1D) (chi square trend analysis p < 0.001). Similar increase in nuclear slug expression was observed in 4/47 mild dysplasia, 3/8 moderate dysplasia and 4/6 severe dysplasia (Figure 1B, C, D respectively) (chi square trend analysis p = 0.001).

We observed similar pattern of Slug expression in ESCC as well. Sixty four of 91 (70.3%) ESCCs showed cytoplasmic localization of Slug in tumor cells as compared to the normal tissues (p < 0.001, OR = 10.0, 95% CI = 4.5 - 23.5, Table 1 and Figure 1E). Notably, significant increase in cytoplasmic Slug expression was observed in ESCCs (70.3%) as compared to dysplasias (39.3%) (p <0.001, OR = 4.2, 95% C.I. = 2.1 - 8.37). Significant increase in nuclear Slug expression was observed in ESCCs (29.7%) as compared to dysplasia (18%) (p = 0.008, OR = 2.9, 95% C.I. = 1.3 - 6.46). In addition to cytoplasm staining, intense Slug nuclear staining was also observed in the of tumor cells in 27 of 91 (29.7%) ESCCs analyzed as compared to the normal tissues (p < 0.001, OR = 1.42, 95% CI = 1.24 - 1.62, Table 1 and Figure 1F). The clinicopathological parameters of ESCCs and their correlation with nuclear/cytoplasmic expression of Slug are shown in Table 1 No immunostaining was observed in ESCC tissue sections used as negative controls where the primary antibody was replaced by isotype specific IgG (Figure 1G), while the positive control showed Slug expression (Figure 1H).

### Slug as a potential diagnostic marker for dysplasia and ESCC

Receiver Operating Characteristic (ROC) analysis was used to determine the potential of Slug overexpression to distinguish dysplasia and ESCC from normal esophageal tissues (Table 2). The values for area-under-the-curve (AUC) were cytoplasmic Slug expression were 0.68 and 0.80 for dysplasia and ESCC respectively (Table 2). The AUC for Slug nuclear staining were 0.63 and 0.68 (Table 2). 

**Table 2:** Correlation of Disease Free Survival with Clinicopathological Parameters and Slug expression: Multivariate Analysis.

**Clinico- pathological Parameter**	**Kaplan Meier Survival analysis Un-adjusted p-value**	**Multivariate Cox regression analysis Adjusted p-value**	**Hazard Ratio ( 95%CI**)
SLUG nuclear	**0.006**	**0.005**	2.269 (1.289-3.996)
SLUG cytoplasm	0.171	0.467	
Nodal Status	0.062	0.427	
Histological differentiation	0.793	0.762	
Tumor Stage	0.209	0.453	

### Slug overexpression as prognostic marker for ESCC

**Figure 2:**
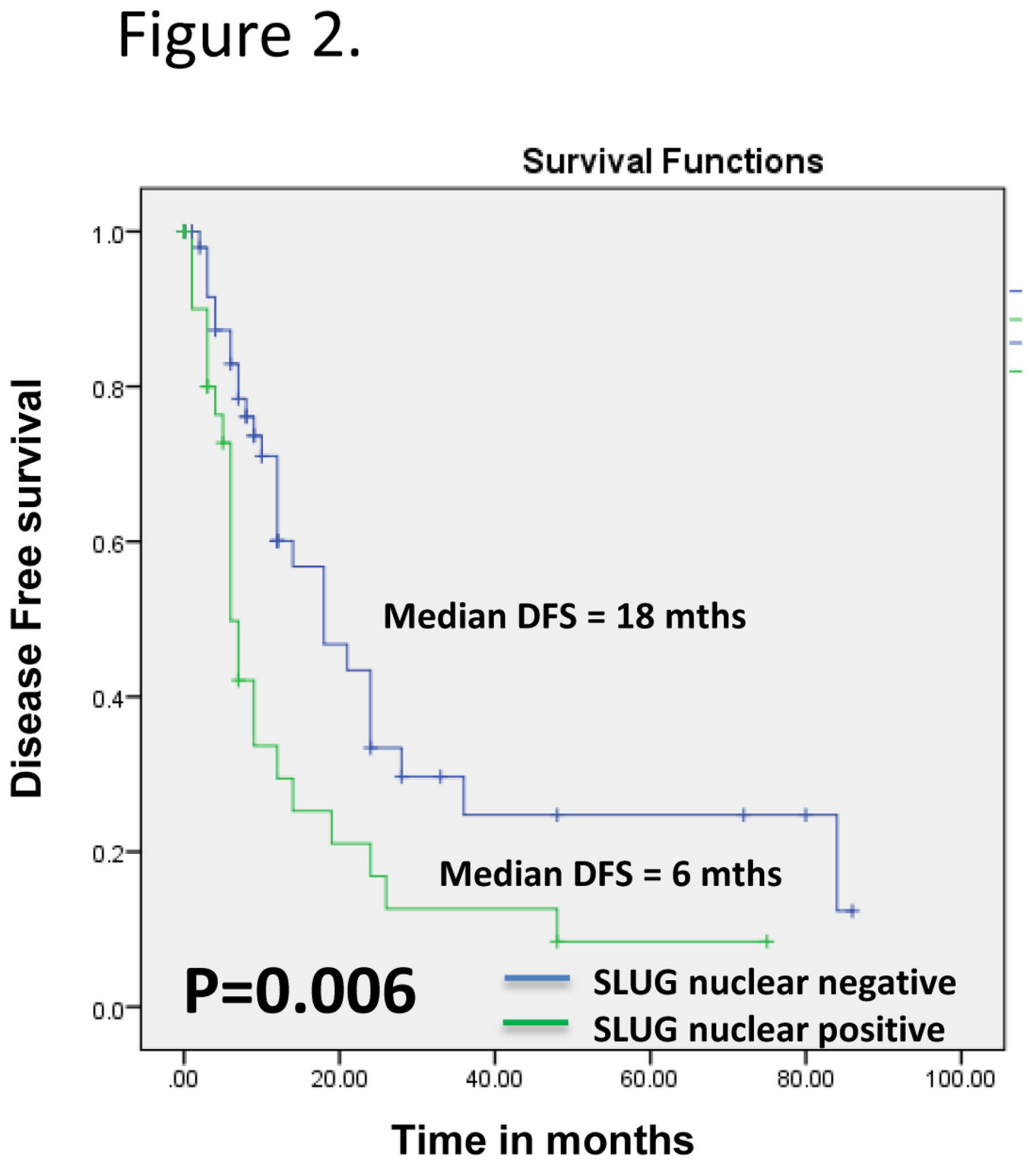
Evaluation of nuclear Slug overexpression as a prognostic marker in ESCC. Kaplan–Meier estimation of cumulative proportion of disease-free survival (DFS): Median time for disease-free survival (DFS; no recurrence/metastasis) in ESCC patients showing nuclear immunostaining of Slug was 6 months, whereas in patients showing no/faint Slug immunostaining in nucleus, median DFS was 18 months (p = 0.006).

 Kaplan-Meier survival analysis showed significantly reduced disease-free survival (median disease free survival (DFS) 6 months; p = 0.006) in ESCC patients with increased nuclear Slug expression as compared to the median DFS of 18 months in patients showing no nuclear Slug immunopositivity (Figure 2). Multivariate Cox regression analysis was carried out to determine the prognostic potential of Slug expression (nuclear/ cytoplasmic) for ESCC in comparison with the other clinical and pathologic parameters including - histological grade, tumor size and nodal status (Table 2). Nuclear Slug expression [p = 0.005, Hazard’s ratio (H.R.) = 2.269, 95% CI = 1.289 - 3.996] emerged as the most significant independent prognostic marker for ESCC.

## Discussion

The salient findings of our study are: (i) significant increase in cytoplasmic Slug expression as early as in esophageal dysplasia and in ESCC in comparison with normal esophageal tissues; and (ii) potential of nuclear Slug overexpression as a marker of poor prognosis of ESCC. Our findings are important in view of the fact that studies on molecular analysis of esophageal dysplasia are limited. This is mainly because these patients do not seek medical attention due to small size of the lesions that do not pose any serious clinical problems and/ or patients often avoid endoscopic examination. Therefore, tissue specimens from early preneoplastic lesions are not readily available for biomarker analysis; consequently, there are no established biomarkers that can be used in clinics routinely in early stages of the disease. Hence, overexpression of Slug observed in dysplastic lesions is an important finding of our study. Furthermore, Slug expression was associated with grade of dysplasia, low grade (mild) dysplasia showing less Slug expression as compared to the high grade (severe) dysplasia. The hallmark of the study was the detection of Slug protein in endoscopic biopsies of esophageal epithelial dysplasia, suggesting its potential for development as an early biomarker. We are cognizant of the fact that limitations of our study are the small size of dysplasia cases investigated and lack of follow-up data of patients with dysplasia. Nevertheless, to our knowledge this is the first study demonstrating overexpression of Slug in early stage prior to development of frank malignancy, as well as in ESCC, that offers an opportunity for early detection and intervention for effective management of this disease, which otherwise has poor prognosis (overall 5-year survival ranges from 15-30%) particularly when detected in late stages. 

The onset of dysplasia is often associated with chronic inflammation and the molecular links between inflammation and pre-malignancy are being intensely pursued; NFκB, a regulator of EMT being one such link. In this context, earlier studies have also reported the role of Slug protein in EMT as a regulator in primary human cancers [26]. Overexpression of Slug is associated with malignant progression of esophageal adenocarcinoma [12], breast cancer [9,27], lung cancer [28], bladder cancer [29]. Slug also promotes tumor invasion in lung adenocarcinoma [30]. EMT is a critical event in the progression toward cancer metastasis. The dissolution of the E-cadherin-mediated adherens junction (AJ) is a key preliminary step in EMT and may occur early or late in the growing epithelial tumor. This is a first step for tumor cells towards stromal invasion, intravasation, extravasation and distant metastasis [31].

In this context, significant increase in cytoplasmic and nuclear Slug expression observed in ESCCs as compared to dysplasia is another important finding of our study. Our findings support recent studies reporting nuclear and cytoplasmic Slug expression in benign pancreatic ductal epithelium, chronic pancreatitis, pancreatic ductal adenocarcinomas and esophageal adenocarcinomas [12,32]. 

Our study showed significance of nuclear Slug expression as a predictor of poor prognosis of ESCC (independent of other clinical and pathological parameters as revealed by Cox regression model). These findings demonstrated the potential of nuclear Slug as a marker for poor prognosis of ESCC. In support of our findings Shioiri et al. [33], reported the association of overexpression of Slug with poor survival in colorectal carcinoma patients.

In conclusion, Slug was shown to be expressed in premalignant lesions, dysplasia, and in frank tumors. Cytoplasmic/nuclear Slug may serve as an early diagnostic marker for ESCC and nuclear Slug as a prognostic marker for disease recurrence for ESCC patients. 

### Ethics statement

The study was approved by All India Institute of Medical Sciences Research Ethics Board, New Delhi, India. Written informed consent was obtained for the acquisition and use of patient tissue samples and anonymized clinical data.
